# Severe Acute Axonal Neuropathy following Treatment with Arsenic Trioxide for Acute Promyelocytic Leukemia: a Case Report

**DOI:** 10.4084/MJHID.2016.023

**Published:** 2016-04-01

**Authors:** Marcus Kühn, Kety Sammartin, Mitja Nabergoj, Fabrizio Vianello

**Affiliations:** Hematology and Immunology Unit, Padua University School of Medicine, via Giustiniani 1, 35128, Padua, Italy

## Abstract

Peripheral neuropathy is a common complication of arsenic toxicity. Symptoms are usually mild and reversible following discontinuation of treatment. A more severe chronic sensorimotor polyneuropathy characterized by distal axonal-loss neuropathy can be seen in chronic arsenic exposure. The clinical course of arsenic neurotoxicity in patients with coexistence of thiamine deficiency is only anecdotally known but this association may potentially lead to severe consequences.

We describe a case of acute irreversible axonal neuropathy in a patient with hidden thiamine deficiency who was treated with a short course of arsenic trioxide for acute promyelocytic leukemia. Thiamine replacement therapy and arsenic trioxide discontinuation were not followed by neurological recovery and severe polyneuropathy persisted at 12-month follow-up.

Thiamine plasma levels should be measured in patients who are candidate to arsenic trioxide therapy. Prophylactic administration of vitamin B1 may be advisable. The appearance of polyneuropathy signs early during the administration of arsenic trioxide should prompt electrodiagnostic testing to rule out a pattern of axonal neuropathy which would need immediate discontinuation of arsenic trioxide.

## Case report

Although polyneuropathy is common following arsenic trioxide therapy of subjects with acute promyelocytic leukemia (APL), symptoms are generally mild and they disappear after completion of arsenic treatment. In November 2014, a 72-year old Caucasian woman was admitted to this Unit with fever and pancytopenia (white blood cell count 0.19 × 10^9^/L, hemoglobin 76 g/L, platelets 41 x10^9^/L). She had a medical history of lobular carcinoma of the left breast treated with mastectomy and chemotherapy in 2012, Hashimoto thyroiditis, arterial hypertension. There was no prior neurological disorder and physical examination was unremarkable. Coagulation tests showed reduced prothrombin time ratio (59%) and normal activated partial thromboplastin time, a slightly reduced fibrinogen (1,4 g/L) and increased D-dimer (8634 μg/L). Bone marrow evaluation was consistent with acute promyelocytic leukemia (APL) according to French-American-British (FAB) classification system. The patient was started on all-trans retinoic acid (45 mg/m^2^) according to protocol APL0406^1^ and, by day 3, arsenic trioxide (ATO) 10 mg/d was added at the time of PML/RAR alpha rearrangement identification by PCR. The clinical course was complicated by differentiation syndrome, which manifested as mild increase of creatinine levels, peripheral edema and pleuro-pericardial effusion. All-trans retinoic acid was therefore stopped on day 12, dexamethasone and furosemide added and the patient was continued on arsenic trioxide.

On day 16, the patient gradually regained kidney function and all-trans retinoic was resumed. Starting on day 22, progressive cognitive impairment, insomnia, slurred speech, spatial and temporal disorientation occurred. As this worsening clinical picture was concomitant to hypernatremia (up to 160 mmol/L, reference values 136–145 mmol/L), neurological symptoms were attributed to sodium-related hyperosmolarity.

Over the following days an impaired level of consciousness and lethargy was noticed. Progressive and appropriate normalization of natriemia did not improve neurological dysfunction.

Additional diagnostic procedures were performed. A CT scan of the brain did not reveal abnormalities related to the acute clinical picture, showing only age-related mild cerebral and cerebellar atrophy. Cerebrospinal fluid showed only mild protein elevation.

Plasma levels of vitamin B1 were low (22 nmol/L, reference values 66–200 nmol/L) and supplementation was started. Over the following 5 days the patient showed a slow but progressive full recovery of cognitive functions.

Unexpectedly, at normalization of cognitive function, a pattern of symmetric peripheral neuropathy involving upper and lower limbs became clinically evident. Physical examination showed that left and right hands were severely weak, particularly the abductor pollicis brevis, with mild strength reduction in the proximal arms. Deep-tendon reflexes were absent. There was bilateral foot drop, and the patient was unable to walk. Strength in the ankle dorsiflexors, extensor hallucis longus, extensor digitorum brevis, and toe flexors was severely compromised. There was no muscle atrophy or fasciculation, and the remainder of the neurologic examination was normal. Electromyography (EMG) showed a severe sensory-motor axonal neuropathy of the upper and lower extremities with a greater reduction of sensory action potential.

Meanwhile on day 28 arsenic trioxide had been stopped according to the therapy protocol. Bone marrow evaluation showed molecular remission. At that time the patient was bedridden because of a severe neurological impairment, so we chose not to administer consolidation therapy as it would have been required according to standard protocol.^1^ The patient was then continued on maintenance therapy with methotrexate and 6-mercaptopurine. Molecular remission was confirmed in bone marrow aspirate at the 13-month follow-up.

Over the following on 6 months from arsenic trioxide discontinuation, the patient regained full proximal muscle strength but with almost complete persistence of marked distal weakness of the hands,ly finger and wrist extensors, weakness of foot dorsiflexors bilaterally and the inability to maintain an upright position. Neurological examination and EMG were substantially unchanged at the 12-month follow-up evaluation.

We believe that occult thiamine deficiency exacerbated arsenic trioxide neurotoxicity causing an unexpected and irreversible distal axonal symmetric neuropathy. This is the first report of acute and irreversible axonal neuropathy in a patient treated with arsenic trioxide with a background of thiamine deficiency. Peripheral neuropathy has long since been associated with the use of arsenic. Several clinical studies have been published on ATO treatment of patients with APL (Table).^2^–^12^ Although details on grade and duration of peripheral neuropathy were not always clearly included, overall grade 3/4 neuropathy accounts for about 0.5% of patients treated with ATO and recovery is always observed. Yip et al reported a case of severe neurotoxicity during arsenic therapy in a subject with APL and occult thiamine deficiency.^13^ However, thiamine administration led to rapid improvement suggesting a major role of thiamine deficiency over arsenic toxicity.

Severe and irreversible arsenic neurotoxicity in the setting of thiamine deficiency can be explained by their common metabolic target. In fact, thiamine deficiency and arsenic exposure severely impair pyruvate dehydrogenase (PDH) activity, an enzyme responsible for converting glucose to energy in high yield. As neural tissues are highly dependent on carbohydrates for energy production and cellular metabolism, it is tempting to speculate a synergic detrimental neuropathic effect leading to the severe toxicity observed in our patient. Of interest, we found electrophysiological findings consistent with acute axonal dysfunction without pattern of demyelination. This type of damage is the classic electrophysiological and histopathological finding in beriberi neuropathy whereas arsenic axonal toxicity characteristically coexists with segmental demyelination.^14^,^15^ An acute pattern of axonal damage has been reported as a manifestation of arsenic poisoning but it has not been observed as a side effect of short term administration of arsenic trioxide at therapeutic dosage.^16^ Thus, arsenic trioxide therapy may lead to axonal loss through the exacerbation of the metabolic damage to the nerve tissue induced by thiamine deficiency. This may have relevance in considering prophylactic administration of thiamine to subjects undergoing arsenic trioxide therapy for APL, a clinical approach already adopted by others^7^ and supported by animal models of antioxidant properties of thiamine in arsenic treated animals.^17^

In conclusion, occult thiamine deficiency may contribute to arsenic toxicity and the combination may cause irreversible axonal neuropathy which must be considered when monitoring APL patients on arsenic trioxide therapy. Prophylactic administration of thiamine may be considered in this setting.

## Figures and Tables

**Table:** Clinical studies reporting neurological adverse effects in APL patients treated with ATO

	No. of cases	All grade N° (%)	Grade 3/4 N° (%)	Clinical course of neuropathy
Soignet et al 2	12	3 (25)	0	-
Niu et al 3	58	6 (10.3)	0	-
Soignet et al 4	40	17 (42.5)	1 (2,5)	FR
Lazo et al 5	12	2	1	I
Raffoux et al 6	20	2	0	-
Shen et al 7	41	0	-	-
Shigeno et al 8	34	10 (29)	0	-
Mathews et al 9	72	14 (19.4)	2 (2.7)	I
Estey et al 10	45	1 (2.2)	NR	NR
Ghavamzadeh et al 11	197	0	-	-
Iland et al 12	124	9 (7.2)	9 (7.2)	NR

* Neurological side effects were dizziness, mood alteration, musculoskeletal pain, or seizure. Only 1 patient had grade 3 peripheral neuropathy during the induction phase. I: improved; FR: Full Recovery; NR: not reported
